# LncRNAs Associated with Neuronal Development and Oncogenesis Are Deregulated in SOD1-G93A Murine Model of Amyotrophic Lateral Sclerosis

**DOI:** 10.3390/biomedicines9070809

**Published:** 2021-07-13

**Authors:** Federica Rey, Stefania Marcuzzo, Silvia Bonanno, Matteo Bordoni, Toniella Giallongo, Claudia Malacarne, Cristina Cereda, Gian Vincenzo Zuccotti, Stephana Carelli

**Affiliations:** 1 Department of Biomedical and Clinical Sciences “L. Sacco”, University of Milan, Via Grassi 74, 20157 Milano, Italy; federica.rey@unimi.it (F.R.); toniella.giallongo@unimi.it (T.G.); gianvincenzo.zuccotti@unimi.it (G.V.Z.); 2 Paediatric Clinical Research Center Fondazione “Romeo ed Enrica Invernizzi”, University of Milano, 20157 Milano, Italy; 3 Neurology IV-Neuroimmunology and Neuromuscular Diseases Unit, Fondazione IRCCS Istituto Neurologico Carlo Besta, Via Celoria 11, 20133 Milan, Italy; stefania.marcuzzo@istituto-besta.it (S.M.); silvia.bonanno@istituto-besta.it (S.B.); claudia.malacarne@istituto-besta.it (C.M.); 4 Centro di Eccellenza Sulle Malattie Neurodegenerative, Dipartimento di Scienze Farmacologiche e Biomolecolari (DiSFeB), Università Degli Studi di Milano, Via Balzaretti 9, 20133 Milano, Italy; matteo.bordoni@unimi.it; 5 PhD Program in Neuroscience, University of Milano-Bicocca, Via Cadore 48, 20900 Monza, Italy; 6 Genomic and Post-Genomic Center, IRCCS Mondino Foundation, 27100 Pavia, Italy; cristina.cereda@mondino.it; 7 Department of Pediatrics, Children’s Hospital “V. Buzzi”, Via Lodovico Castelvetro 32, 20154 Milano, Italy

**Keywords:** Amyotrophic Lateral Sclerosis, lncRNAs, SOD1-G93A, neurodegenerative diseases, neural development, oncogenes, linc-p21, pre-symptomatic ALS, familial ALS, biomarkers

## Abstract

Amyotrophic Lateral Sclerosis (ALS) is a devastating neurodegenerative disease caused in 10% of cases by inherited mutations considered “familial”. An ever-increasing amount of evidence is showing a fundamental role for RNA metabolism in ALS pathogenesis, and long non-coding RNAs (lncRNAs) appear to play a role in ALS development. Here, we aim to investigate the expression of a panel of lncRNAs (linc-Enc1, linc–Brn1a, linc–Brn1b, linc-p21, Hottip, Tug1, Eldrr, and Fendrr) which could be implicated in early phases of ALS. Via Real-Time PCR, we assessed their expression in a murine familial model of ALS (SOD1-G93A mouse) in brain and spinal cord areas of SOD1-G93A mice in comparison with that of B6.SJL control mice, in asymptomatic (week 8) and late-stage disease (week 18). We highlighted a specific area and pathogenetic-stage deregulation in each lncRNA, with linc-p21 being deregulated in all analyzed tissues. Moreover, we analyzed the expression of their human homologues in SH-SY5Y-SOD1-WT and SH-SY5Y-SOD1-G93A, observing a profound alteration in their expression. Interestingly, the lncRNAs expression in our ALS models often resulted opposite to that observed for the lncRNAs in cancer. These evidences suggest that lncRNAs could be novel disease-modifying agents, biomarkers, or pathways affected by ALS neurodegeneration.

## 1. Introduction

Amyotrophic Lateral Sclerosis (ALS) is a devastating neurodegenerative disease characterized by the progressive loss of upper and lower motor neurons, which leads to muscle atrophy, paralysis, and ultimately death two to five years after the first diagnosis [1]. Approximately 10% of ALS cases can be considered “familial” (fALS), meaning they are caused by a specific mutation in ALS genes transmitted within families [2]. The SOD1 gene was the first identified ALS gene [3], and to this day approximately 18.9% of fALS cases and 1.2% of sporadic ALS (sALS) cases can be attributed to mutations in this gene [2]. As mutations in this gene represent indeed almost 20% of familial cases, murine models carrying a SOD1-gene mutation, such as the SOD1-G93A mice, can provide insights into ALS pathogenesis, as they present an ALS-like phenotype which includes mitochondrial dysfunctions, SOD1 aggregation, motor neuron death, and paralysis [2].

Even though more and more evidence is arising pertaining to new disease-causing mechanisms, we are still far from a clear characterization of ALS’s pathogenesis. With this in mind there is still a crucial need to investigate novel implicated molecular mechanisms. Mounting evidences recommend a fundamental role for RNA metabolism in ALS pathogenesis [4,5,6], supported by the most recent identification of mutations in TAR DNA binding protein 43 (TDP-43) and Fused-In-Sarcoma (FUS) genes as pathological hallmarks of the disease [2,7]. Moreover, non-coding RNAs are also appearing to be strongly involved in ALS, with an implication for both long non-coding RNAs (lncRNAs, >200 bp) and small non-coding RNAs such as miRNAs (<200 bps) [5,7,8,9,10]. In this context, there is a significant interest in investigating lncRNAs in ALS, as this class of molecules, in terms of functions and mechanisms of action, is widely unknown and still uncharacterized, and they have been found to present a significant deregulation in biological samples (PBMCs) of disease affected patients [5,8]. Indeed, through RNA-seq in PBMCs of sporadic ALS patients, a total of 293 differentially expressed (DE) lncRNAs was found, as opposed to only 87 differentially expressed coding genes [8]. It has been suggested that lncRNAs could be implicated in early phases of the disease, and indeed, neuronal proliferation and early development processes play a role in ALS. Studies demonstrated how in SOD1-G93A mice stem progenitor cells show a higher proliferation and differentiation towards the neuronal lineage, differing from stem progenitor cells obtained from WT mice [11,12]. Moreover, proliferation in neural stem progenitor cells has been demonstrated in the subventricular zone of ALS patients, suggesting that there is an increased neural proliferation that takes place during disease pathogenesis [13].

Recently a panel of lncRNAs (*linc-Enc1*, *linc–Brn1a*, *linc–Brn1b*, *linc-p21*, *Hottip*, *Tug1*, *Eldrr*, and *Fendrr*) was identified to be implicated in murine development and subventricular zone (SVZ) neural stem cells differentiation [14,15]. In particular, *linc–Brn1a*, *linc–Brn1b*, *Eldrr*, *Fendrr*, *linc-p21*, and *Tug1* are among those with a strong expression in Embryonic stem-derived Neural Stem Cells (ES-NSCs), implicating them in the biology and cellular function of ES-NSCs [14]. Interestingly, all these lncRNAs are also found deregulated in numerous tumors, and this could be an interesting prospect as oncogenic alterations often present an opposite mechanism to those observed in neurodegeneration [16,17,18,19,20,21,22,23,24,25,26]. As they present these intriguing characteristics, it would be interesting to investigate if these lncRNAs are deregulated in neurodegenerative diseases. Furthermore, as previously stated, they present a role not only in neural stem cells but also in the adult age, due to their involvement in tumor development and even neurodegenerative diseases, as linc-p21 has been found implicated in Parkinson’s Disease [27]. It would thus be of interest to investigate their regulation during murine aging, in both healthy and diseased mice.

To this end, the aim of this work was to investigate a panel of lncRNAs (*linc-Enc1*, *linc–Brn1a*, *linc–Brn1b*, *linc-p21*, *Hottip*, *Tug1*, *Eldrr*, and *Fendrr*) implicated in both murine development and oncogenesis, in a murine familial model of ALS (the SOD1-G93A mouse). Specifically, we analyzed their expression in brain and spinal cord areas of SOD1-G93A mice in comparison with that of B6.SJL control mice, in asymptomatic phase of the disease (week 8 of age) and late-stage disease (week 18 of age). As none of these lncRNAs have ever been previously correlated with ALS pathogenesis, an evidence of their implication could lead to their identification as possible disease-modifying agents, biomarkers, or secondary effects of the ALS neurodegenerative process.

## 2. Materials and Methods

### 2.1. Animals

Transgenic SOD1-G93A (B6SJL-Tg(SOD1*G93A)1Gur) and control B6.SJL mice were purchased from Charles River, Inc. (Wilmington, MA, USA), maintained and bred at the animal house of the Fondazione IRCCS Istituto Neurologico Carlo Besta, in compliance with institutional guidelines, international regulations (EEC Council Directive 86/609) and project approvals from the Italian Ministry of Health (ref. IMP183/2018-PR). Transgenic SOD1-G93A progenies were identified by real-time PCR amplification of the mutant human SOD1 gene, respectively, as previously described [28]. All the SOD1-G93A animals included in the study had more than 27 mutant SOD1 copies. By motor score and PaGE test analysis, the first clinical signs of motor neuron disease in SOD1-G93A were observed from week 12 (disease onset) with hind limb tremors and reduced muscular strength. Moreover, from week 13 the SOD1-G93A mice presented a significant decreased of body weight compared to controls. As disease progressed, from weeks 15 (symptomatic phase) to 18 (late-stage disease), these pathological alterations became more evident with significant muscle atrophy, as previously described [28]. SOD1-G93A mice were sacrificed by exposure to CO_2_ at week 8 (pre-symptomatic) or week 18 (late-stage disease). Males were used in all experiments. They were sacrificed by exposure to CO_2_ at week 8 (pre-symptomatic) or week 18 (late-stage disease).

### 2.2. Isolation of Specific Brain and Spinal Cord Regions

Each brain region was identified with the aid of the coordinates shown in the mouse brain atlas. We manually dissected out the brain regions of interest using a dissecting microscope and scalpel, as previously described [9]. Specifically, we first cut a 2 mm-thick slice including the prefrontal cortex. Next, we cut a 3 mm-thick slice containing the primary motor cortex, secondary motor cortex, and dorsal and ventral parts of the SVZ and striatum. A successive coronal section of 2 mm-thick contained the hippocampus, readily identified by visual inspection. We next obtained a consecutive coronal section, the brainstem motor nuclei and the trigeminal nucleus. A 1 mm-thick slice was taken, which itself contained the evident fourth ventricle; below this continued the area of the brainstem motor nuclei, which was also separated using a scalpel and united with the material of the previous section to collect the mesencephalon region. By clear visual inspection, a final 2 mm-thick slice that included cerebellum was cut.

Total spinal cord was manually dissected out and cut into cervical, thoracic, and lumbar sections. Each spinal cord region was identified with the aid coordinates shown in the mouse spinal cord atlas; specifically, we obtained the cervical (C2–C6), thoracic (T2–T12), and lumbar (L2–L5) sections.

### 2.3. Cell Culture

Human neuroblastoma SH-SY5Y cells (ATCC), SH-SY5Y-SOD1-WT, and SH-SY5Y-SOD1-G93A were grown in DMEM Low Glucose (Gibco) supplemented with 15% of Fetal Bovine Serum (Euroclone), 1% L-glutamine Gibco) and 1% penicillin/streptomycin (Gibco) at 37 °C in a 5% CO_2_ atmosphere. SH-SY5Y-SOD1-G93A and SH-SY5Y-SOD1-WT were previously obtained by transfection with the expression plasmid pRc/CMV (Invitrogen, CA, USA) containing cDNAs coding for the SOD1 protein [29]. SH-SY5Y cells were subsequently stably transfected with lipofectamine 2000 transfection reagent (Invitrogen) and kept in OPTIMEM (Gibco). After 48 h incubation, the culture medium was changed, and cells were cultured with selective medium containing gentamicin-disulfate G418 (Gibco). The SH-SY5Y cells, SH-SY5Y-SOD1-WT and SH-SY5Y-SOD1-G93A were monitored for growth capabilities. As previously reported, cell lines were seeded on 12-well plates at a density of 5 × 10^5^ cells/well in complete culture medium. After 24, 48 h, and 72 h cells were harvested using trypsin (200 µL/well) and the number of live cells were counted by Trypan blue exclusion test [29].

### 2.4. RNA Extraction

Total RNA was isolated from the samples of interest using TRIzol Reagent (Invitrogen, Carlsbad, CA, USA) following standard protocol. RNA quality was assessed using a spectrophotometer (NANOPhotometer^®^ NP80, IMPLEN, Westlake Village, CA, USA).

### 2.5. Real-Time PCR

Total RNA (1000 ng) was reverse transcribed using iScript cDNA Synthesis Kit (Bio-Rad, Hercules, CA, USA) according to the manufacturer’s instructions. Using gene sequences available from NCBI for target genes (http://www.ncbi.nlm.nih.gov/nucleotide (accessed on 29 May 2020)) PCR oligonucleotide primers for target genes were selected and primers are reported in Table S1. This was done with the NCBI’s Primer-BLAST tool. Real-time PCR was performed with StepOnePlusTM Real-Time PCR System (Thermo Fisher, Waltham, MA, USA) using SSOSYBR Green Supermix (Bio-Rad, Hercules, CA, USA). Genes were quantified in triplicates, GAPDH was used as housekeeping gene for mouse samples whereas 18S was used for studies in SH-SY5Y. Gene expression was calculated using the 2^−ΔΔCt^ method.

## 3. Results

### 3.1. Linc-Brn1a and Linc-Brn1b Present Altered Expression in CNS Areas of SOD1-G93A Mice

In this research we assessed the expression of a panel of lncRNAs in SOD1-G93A mice at 8 or 18W of age. All the SOD1-G93A animals included in the study had more than 27 mutant SOD1 copies. By motor score and PaGE test analysis, the first clinical signs of motor neuron disease in SOD1-G93A were observed from week 12 (disease onset) with hind limb tremors and reduced muscular strength. Moreover, from week 13 the SOD1-G93A mice presented a significant decrease in body weight compared to controls. As disease progressed, from weeks 15 (symptomatic phase) to 18 (late- stage disease), these pathological alterations became more evident with significant muscle atrophy, as previously described [28].

**Figure 1:**
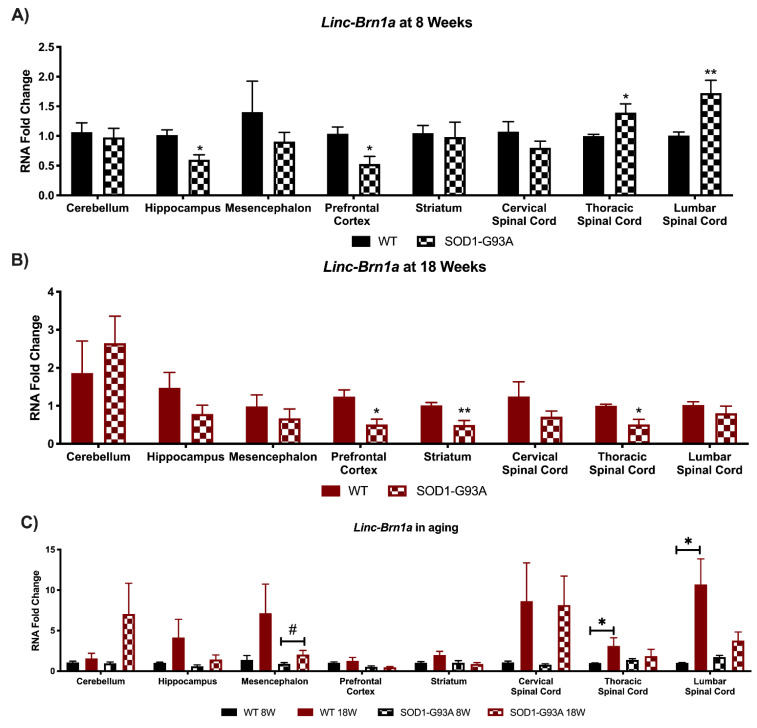
Global perturbations found in the expression of *Linc-Brn1a* in CNS areas of SOD1-G93A mice. RNAs expression levels were evaluated by Real Time-PCR in the different conditions. Data are expressed as mean ± SEM. (**A**) *Linc-Brn1a* expression in 8 weeks old WT (*n* = 6) mice versus 8 weeks old SOD1-G93A mice (*n* = 6). * *p* < 0.05, ** *p* < 0.01 vs. WT. (**B**) *Linc-Brn1a* expression in 18 weeks old WT (*n* = 6) mice versus 18 weeks old SOD1-G93A mice (*n* = 6). * *p* < 0.05, ** *p* < 0.01 vs. WT. (**C**) *Linc-Brn1a* expression in 8 weeks old WT mice versus 18 weeks old WT (*n* = 6) and 8 weeks old SOD1-G93A mice versus 18 weeks old SOD1-G93A mice (*n* = 6). * *p* < 0.05 vs. WT 8W, # *p* < 0.05 vs. SOD1-G93A 8W.

**Figure 2:**
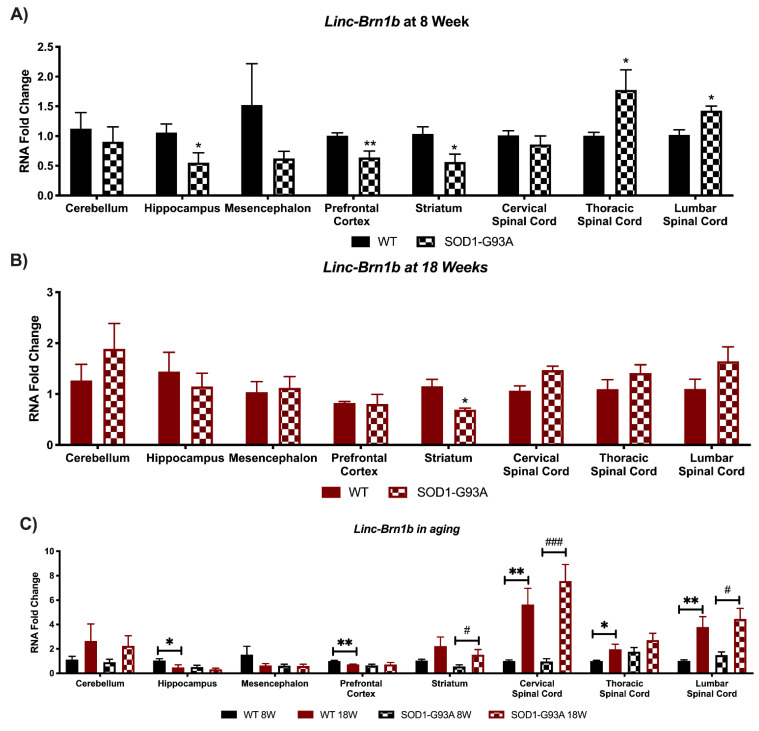
Global perturbations found in the expression of *Linc-Brn1b* in CNS areas of SOD1-G93A mice. RNA expression levels were evaluated by Real Time-PCR in the different conditions. Data are expressed as mean ± SEM. (**A**) *Linc-Brn1b* expression in 8 weeks old WT (*n* = 6) mice versus 8 weeks old SOD1-G93A mice (*n* = 6). * *p* < 0.05, ** *p* < 0.01 vs. WT. (**B**) *Linc-Brn1b* expression in 18 weeks old WT (*n* = 6) mice versus 18 weeks old SOD1-G93A mice (*n* = 6). * *p* < 0.05 vs. WT. (**C**) *Linc-Brn1b* expression in 8 weeks old WT mice versus 18 weeks old WT (*n* = 6) and 8 weeks old SOD1-G93A mice versus 18 weeks old SOD1-G93A mice (*n* = 6). * *p* < 0.05, ** *p* < 0.01 vs. WT 8W, # *p* < 0.05, ### *p* < 0.001 vs. SOD1-G93A 8W.

*Linc-Brn1a* and *linc-Brn1b* are two lncRNAs mainly known for their role in cortical development and stemness maintenance [14,15,30]. Their expression was analyzed in both pre-symptomatic (8W) and symptomatic (18W) SOD1-G93A mice versus controls in the specific brain regions including cerebellum, hippocampus, mesencephalon, prefrontal cortex, and in the spinal cord areas, which are the cervical, thoracic, and lumbar spinal cord regions (Figure 1 and Figure 2). The deregulation in their expression levels is predominant in pre-symptomatic mice, suggesting a possible implication for them in early phases of the disease. Specifically, *linc-Brn1a* resulted significantly decreased in the hippocampus and prefrontal cortex of SOD1-G93A brains, whilst a significant increase was evident in the thoracic and lumbar spinal cord at 8W (Figure 1A). This is relevant as at 8W mice are pre-symptomatic and closer to the development stage. At 18 weeks, *linc-Brn1a* resulted in downregulated prefrontal cortex and striatum, whereas a downregulation was in this case observed in the thoracic spinal cord (Figure 1B). When looking at the lncRNA expression during the mice aging process (8W versus 18W of age) *linc-Brn1a* appeared to increase in almost all areas of the 18W WT mice, with a specific significance in the thoracic and lumbar spinal cord (Figure 1C).

For *linc-Brn1b* in 8W SOD1-G93A mice compared to controls, there was a significant down-regulation in the hippocampus, prefrontal cortex, and striatum, whereas it results significantly upregulated in the thoracic and lumbar spinal cord (Figure 2A). On the contrary, linc-Brn1b expression resulted in most areas like that of wild-type mice in 18W ALS mice, except for the striatum where *linc-Brn1b* was decreased (Figure 2B). *Linc-Brn1b* expression varies significantly in multiple areas of aged mice, with a significant increase in all areas of the spinal cord and in the striatum, and a significant decrease in the prefrontal cortex and hippocampus of WT mice (Figure 2C). Overall, for *linc-Brn1a* and *linc-Brn1b* the most significant changes are present in pre-symptomatic G93A mice (Figure 1A and Figure 2A), and this could be due to the fact that as they are mainly implicated at the developmental stage, and this could be the period when they are most influenced by (or influence the most) ALS pathology. Even so, a significant increase in their expression is detected in the spinal cord during aging (Figure 1C and Figure 2C).

### 3.2. Linc-p21 Presents with Altered Expression in All CNS Areas of Symptomatic SOD1-G93A Mice

**Figure 3:**
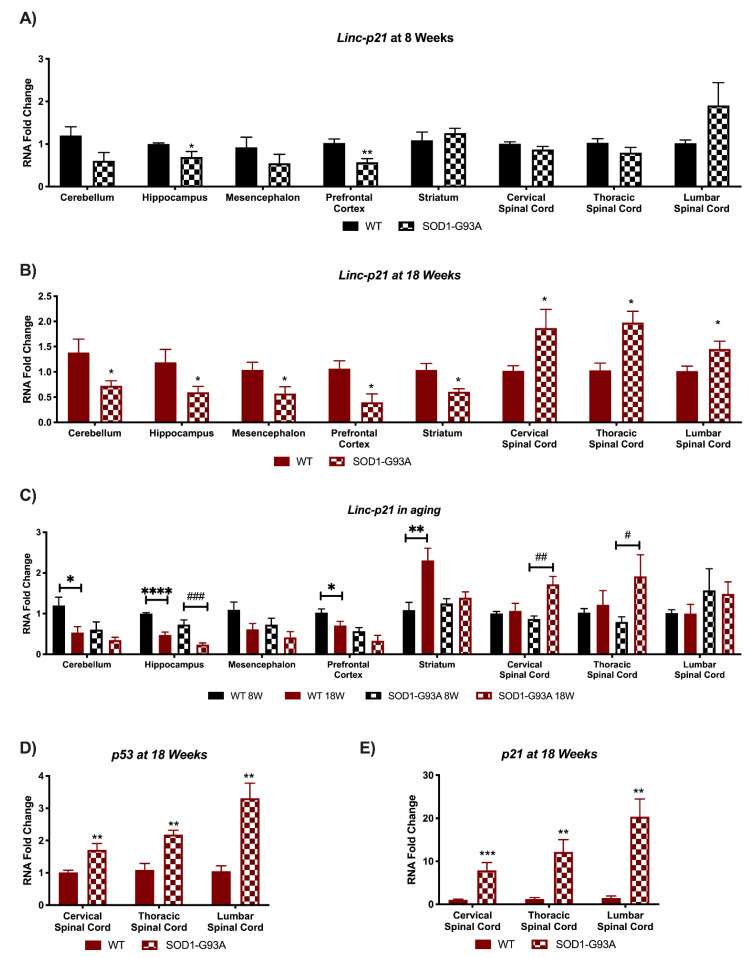
*Linc-p21* is profoundly deregulated in SOD1-G93A mouse. RNAs expression levels were evaluated by Real Time-PCR in the different conditions. Data are expressed as mean ± SEM. (**A**) *Linc-p21* expression in 8 weeks old WT (*n* = 6) mice versus 8 weeks old SOD1-G93A mice (*n* = 6). * *p* < 0.05, ** *p* < 0.01 vs. WT. (**B**) *Linc-p21* expression in 18 weeks old WT (*n* = 6) mice versus 18 weeks old SOD1-G93A mice (*n* = 6). * *p* < 0.05 vs. WT. (**C**) *Linc-p21* expression in 8 weeks old WT mice versus 18 weeks old WT (*n* = 6) and 8 weeks old SOD1-G93A mice versus 18 weeks old SOD1-G93A mice (*n* = 6). * *p* < 0.05, ** *p* < 0.01, **** *p* < 0.0001 vs. WT 8W, # *p* < 0.05, ## *p* < 0.01, ### *p* < 0.001 vs. SOD1-G93A 8W. (**D**) *p53* expression in 18 weeks old WT (*n* = 6) mice versus 8 weeks old SOD1-G93A mice (*n* = 6) spinal cord areas. ** *p* < 0.01 vs. WT. (**E**) *p21* expression in 18 weeks old WT (*n* = 6) mice versus 18 weeks old SOD1-G93A mice (*n* = 6) spinal cord areas. ** *p* < 0.01, *** *p* < 0.001 vs. WT.

*Linc-p21* plays an important role in regulating p53 signaling, cell-cycle, and tumor suppression [31,32]. We found that *linc-p21* expression decreases during neural stem cells differentiation [15]. The expression of *linc-p21* in CNS of SOD1-G93A mice at different stages of the pathology is reported in Figure 3. In the early, pre-symptomatic stage (8W), linc-p21 expression results significantly decreased in the hippocampus and prefrontal cortex in SOD1-G93A mice (Figure 3A). Even so, the most relevant perturbation appears at 18W. Remarkably, *linc-p21* appears as significantly deregulated in all analyzed areas, suggesting a strong implication for this lncRNA in ALS (Figure 3B). Interestingly, *linc-p21* results deregulated in an opposite manner in central brain areas as opposed to the spinal cord, the structure mainly affected by the disease pathology. Specifically, in SOD1-G93A expressing mice *linc-p21* results significantly down-regulated in cerebellum, hippocampus, mesencephalon, prefrontal cortex, and striatum, whereas it results significantly upregulated in cervical, thoracic, and lumbar spinal cord (Figure 3B). Moreover, *Linc-p21* expression appears to be changed at different stages of the mouse lifespan. There is a significant decrease in WT mice from 8 to 18 weeks of age in the cerebellum and prefrontal cortex, and this decrease is extremely significant and present also in the SOD1-G93A mice in the hippocampus area (Figure 3C). In SOD1-G93A mice, *linc-p21* results significantly upregulated in the cervical and thoracic spinal cord, as opposed to 8W vs. 18W control mice (Figure 3C). Possibly this aberrant increase in expression could be responsible of the lncRNAs deregulation in transgenic mice, whilst in the lumbar spinal cord of SOD1-G93A mice *linc-p21* is more expressed even at 8W. As p53 induces *linc-p21* expression, which subsequently induces the expression of p21 [32], these two genes were also analyzed in the spinal cord areas of G93A mice at 18W of age (Figure 3D,E). Remarkably, both the expression of *p53* (Figure 3D) and *p21* (Figure 3E) resulted in significantly increased SOD1-G93A mice in all three areas, highlighting a deregulation of this pathway in the ALS symptomatic animal model.

### 3.3. Tug1 Expression Is Deregulated in Prefrontal Cortex and Lumbar Spinal Cord of Pre- and Symptomatic SOD1-G93A Mice

**Figure 4:**
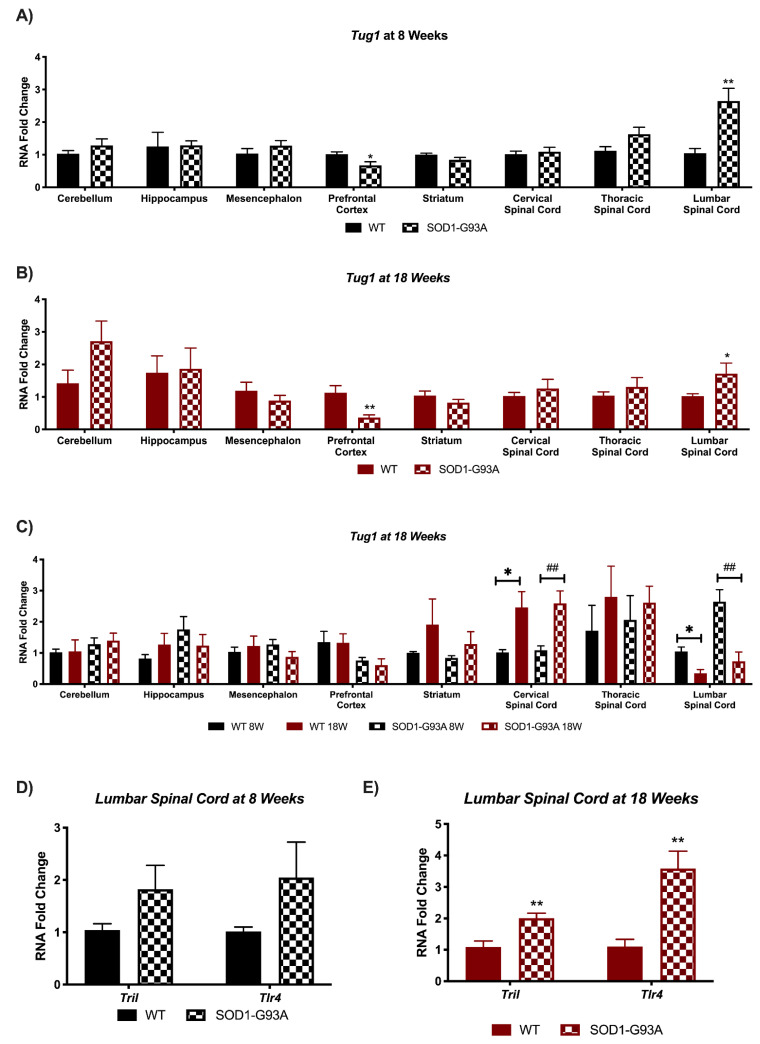
*Tug1* expression is deregulated in spinal cord of SOD1-G93A mice with implications for neuroinflammation processes. RNAs expression levels were evaluated by Real Time-PCR in the different conditions. Data are expressed as mean ± SEM. (**A**) *Tug1* expression in 8 weeks old WT (*n* = 6) mice versus 8 weeks old SOD1-G93A mice (*n* = 6). * *p* < 0.05, ** *p* < 0.01 vs. WT. (**B**) *Tug1* expression in 18 weeks old WT (*n* = 6) mice versus 18 weeks old SOD1-G93A mice (*n* = 6). * *p* < 0.05, ** *p* < 0.01 vs. WT. (**C**) *Tug1* expression in 8 weeks old WT mice versus 18 weeks old WT (*n* = 6) and 8 weeks old SOD1-G93A mice versus 18 weeks old SOD1-G93A mice (*n* = 6). * *p* < 0.05 vs. WT 8W, ## *p* < 0.01 vs. SOD1-G93A 8W. (**D**) *Tril* and *Tlr4* expression in 8 weeks old WT (*n* = 5) mice versus 8 weeks old SOD1-G93A mice (*n* = 5) in lumbar spinal cord. (**E**) *Tril* and *Tlr4* expression in 18 weeks old WT (*n* = 6) mice versus 18 weeks old SOD1-G93A mice (*n* = 6) lumbar spinal cord. ** *p* < 0.01 vs. WT.

*Tug1* is a lncRNA with multiple functions, including a role in spinal cord injury, as it was found to inhibit the expression of *Tril/Tlr4* inflammatory signaling in the spinal cord [33]. *Tug1* was found to have a significantly decreased expression in prefrontal cortex, and, interestingly, a significantly up-regulated expression in the lumbar spinal cord area, the one most affected by ALS, at both the early pre-symptomatic stage at 8 weeks (Figure 4A) and symptomatic stage at 18Weeks (Figure 4B). *Tug1* expression in the brain areas of mice does not vary significantly during aging in the WT nor the SOD1-G93A mice (Figure 4C). On the contrary, an alteration is present in the spinal cord, with a significant increase at 18 weeks for both WT and SOD1-G93A mice in the cervical spinal cord, and a significant decrease in the lumbar spinal cord (Figure 4C). As downregulation of *Tug1* was found to inhibit the expression of *Tril/Tlr4* inflammatory signaling in spinal cords [33], we analyzed whether the opposite mechanism was mediated by its increase in the lumbar spinal cord in ALS (Figure 4D,E). Indeed, we found an increased gene expression already in the pre-symptomatic G93A mice (Figure 4D), which became significant in the late stage of the disease (Figure 4E).

### 3.4. Expression Analysis of Hottip, Eldrr, Linc-Enc1, Fendrr Reveals Global Perturbation in SOD1-G93A Mice

**Figure 5:**
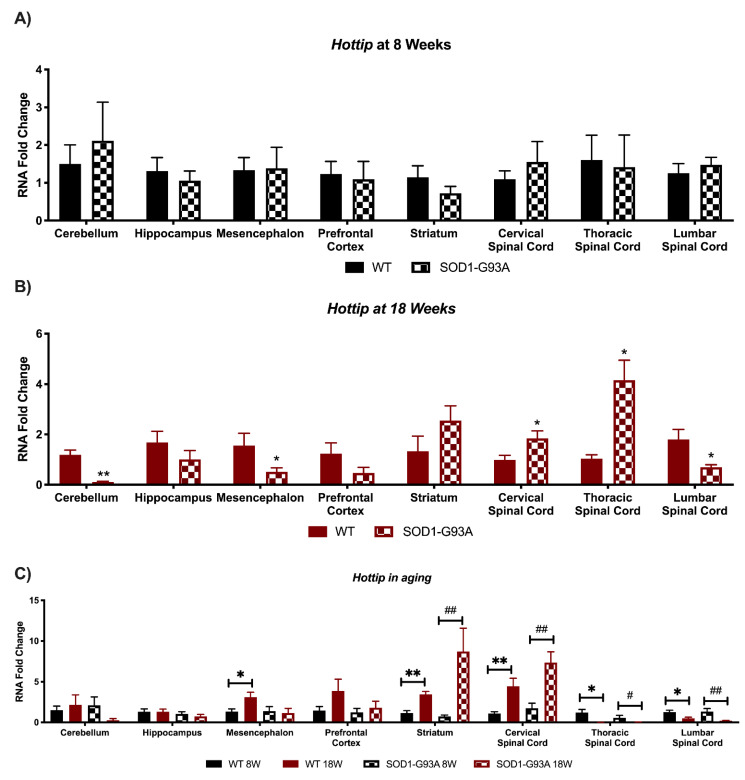
Alterations in *Hottip* expression in SOD1-G93A mice and during murine aging. RNAs expression levels were evaluated by Real Time-PCR in the different conditions. Data are expressed as mean ± SEM. (**A**) *Hottip* expression in 8 weeks old WT (*n* = 6) mice versus 8 weeks old SOD1-G93A mice (*n* = 6). (**B**) *Hottip* expression in 18 weeks old WT (*n* = 6) mice versus 18 weeks old SOD1-G93A mice (*n* = 6). * *p* < 0.05, ** *p* < 0.01 vs. WT. (**C**) *Hottip* expression in 8 weeks old WT mice versus 18 weeks old WT (*n* = 6) and 8 weeks old SOD1-G93A mice versus 18 weeks old SOD1-G93A mice (*n* = 6). * *p* < 0.05, ** *p* < 0.01 vs. WT 8W, # *p* < 0.05, ## *p* < 0.01 vs. SOD1-G93A 8W.

**Figure 6:**
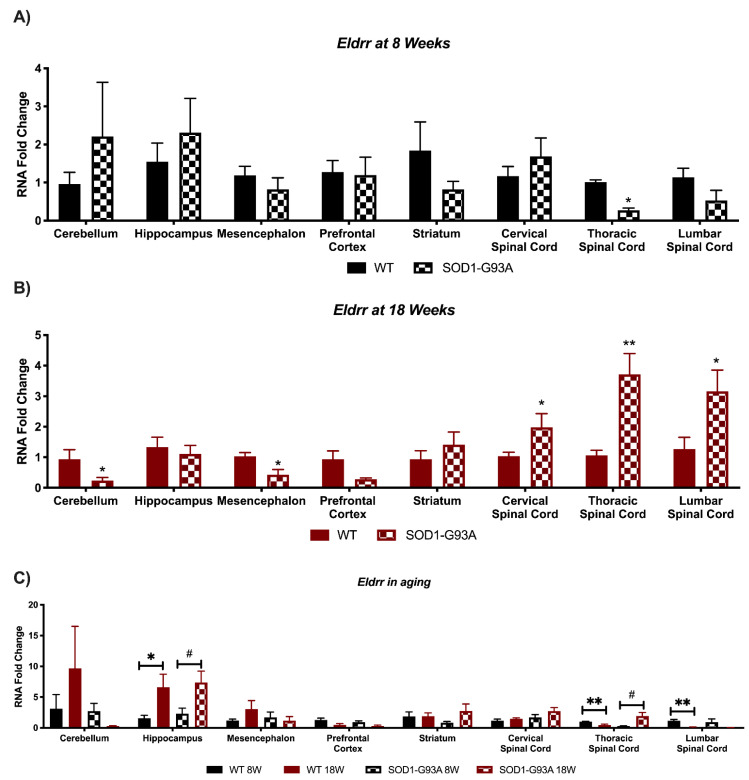
Alterations in *Eldrr* expression in SOD1-G93A mice and during murine development. RNAs expression levels were evaluated by Real Time-PCR in the different conditions. Data are expressed as mean ± SEM. (**A**) *Eldrr* expression in 8 weeks old WT (*n* = 6) mice versus 8 weeks old SOD1-G93A mice (*n* = 6). * *p* < 0.05 vs. WT. (**B**) *Eldrr* expression in 18 weeks old WT (*n* = 6) mice versus 18 weeks old SOD1-G93A mice (*n* = 6). * *p* < 0.05, ** *p* < 0.01 vs. WT. (**C**) *Eldrr* expression in 8 weeks old WT mice versus 18 weeks old WT (*n* = 6) and 8 weeks old SOD1-G93A mice versus 18 weeks old SOD1-G93A mice (*n* = 6). * *p* < 0.05, ** *p* < 0.01 vs. WT 8W, # *p* < 0.05 vs. SOD1-G93A 8W.

**Figure 7:**
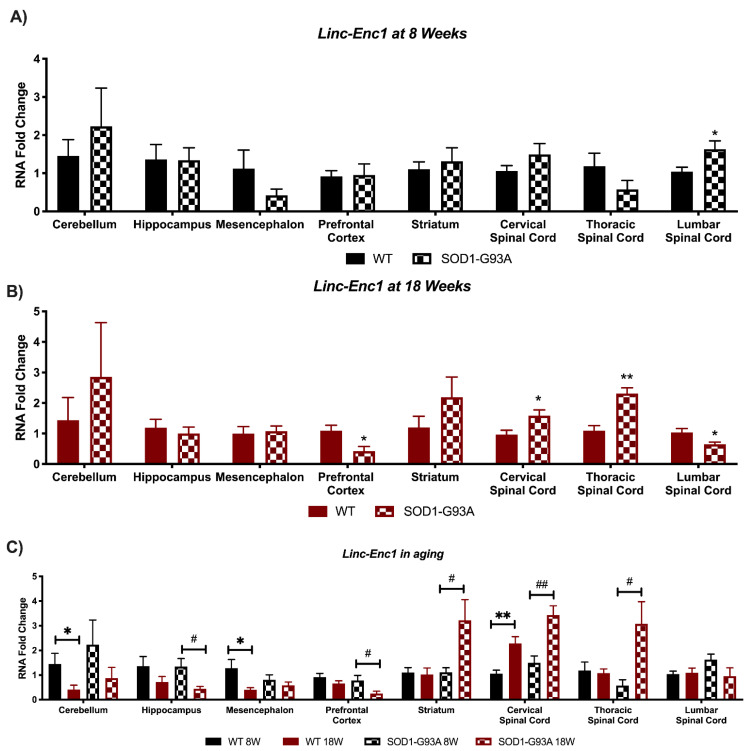
Analysis of *Linc-Enc1* expression shows strong perturbations in SOD1-G93A mice. RNAs expression levels were evaluated by Real Time-PCR in the different conditions. Data are expressed as mean ± SEM. (**A**) *Linc-Enc1* expression in 8 weeks old WT (*n* = 6) mice versus 8 weeks old SOD1-G93A mice (*n* = 6). * *p* < 0.05 vs. WT. (**B**) *Linc-Enc1* expression in 18 weeks old WT (*n* = 6) mice versus 8 weeks old SOD1-G93A mice (*n* = 6). * *p* < 0.05, ** *p* < 0.01 vs. WT. (**C**) *Linc-Enc1* expression in 8 weeks old WT mice versus 18 weeks old WT (*n* = 6) and 8 weeks old SOD1-G93A mice versus 18 weeks old SOD1-G93A mice (*n* = 6). * *p* < 0.05, ** *p* < 0.01 vs. WT 8W, # *p* < 0.05, ## *p* < 0.01 vs. SOD1-G93A 8W.

**Figure 8:**
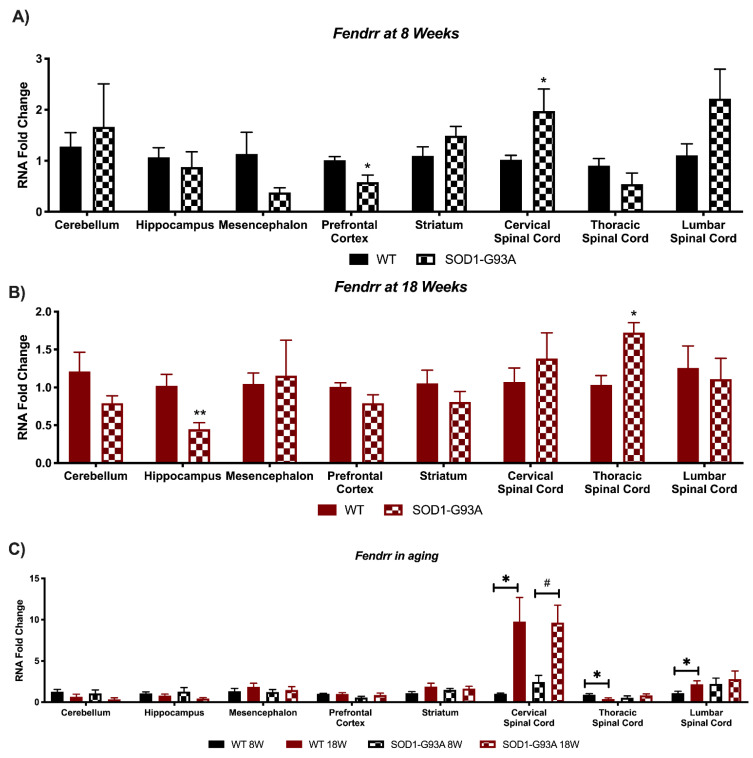
Analysis of *Fendrr* expression shows strong perturbations in SOD1-G93A mice. RNAs expression levels were evaluated by Real Time-PCR in the different conditions. Data are expressed as mean ± SEM. (**A**) *Fendrr* expression in 8 weeks old WT (*n* = 6) mice versus 8 weeks old SOD1-G93A mice (*n* = 6). * *p* < 0.05 vs. WT. (**B**) *Fendrr* expression in 18 weeks old WT (*n* = 6) mice versus 8 weeks old SOD1-G93A mice (*n* = 6). * *p* < 0.05, ** *p* < 0.01 vs. WT. (**C**) *Fendrr* expression in 8 weeks old WT mice versus 18 weeks old WT (*n* = 6) and 8 weeks old SOD1-G93A mice versus 18 weeks old SOD1-G93A mice (*n* = 6). * *p* < 0.05 vs. WT 8W, # *p* < 0.05 vs. SOD1-G93A 8W.

We next decided to assess the expression of four more lncRNAs described as involved in both neural stem cells differentiation [15]: *Hottip, Eldrr, Linc-Enc1*, and *Fendrr* (Figure 5, Figure 6, Figure 7 and Figure 8). *Hottip* presented no significant deregulations in pre-symptomatic mice (Figure 5A) but resulted in perturbation in many areas at 18 weeks in G93A mice (Figure 5B), suggesting it is only affected at later stages of the disease. Specifically, this lncRNA resulted in significant down-regulation in the cerebellum, mesencephalon, and lumbar spinal cord, whereas it was up-regulated in the cervical and thoracic spinal cord (Figure 5B). Moreover, when looking at *Hottip*, a change in its expression was found as the mice aged in multiple areas. Specifically, in WT mice, the lncRNA increased in the mesencephalon, striatum, and cervical spinal cord whereas it decreased in the thoracic and lumbar spinal cord (Figure 5C). Similarly, in SOD1-G93A mice *Hottip* increased in the striatum and cervical spinal cord, and it decreased in the thoracic and lumbar spinal cord. (Figure 5C).

*Eldrr* resulted significantly deregulated in pre-symptomatic mice only in the thoracic spinal cord area, whilst no significant perturbation was found in the other areas (Figure 6A). On the contrary, it resulted in down-regulation in the cerebellum and mesencephalon, and up-regulation in all three areas of the spinal cord, in symptomatic mice (Figure 6B). The most significant deregulations in the aging brain were found in the hippocampus, where *Eldrr* is upregulated in both WT and SOD1-G93A mice (Figure 6C). An interesting trend was found in the thoracic and lumbar spinal cord, where the lncRNA decreased in WT mice, but increased in SOD1-G93A mice (Figure 6C).

*Linc-Enc1* presented a significant up-regulation in the lumbar spinal cord at 8 weeks of SOD1-G93A mice (Figure 7A), but this trend was inverted at 18 weeks when it resulted in down-regulation in the lumbar spinal cord and prefrontal cortex. Moreover, an opposite regulation in the cervical and thoracic spinal cord was found (Figure 7B). Differences in the expression were also found when looking at *linc-Enc1* during mice aging. Physiologically, the lncRNA was found to decrease in the cerebellum and in the mesencephalon, whilst an increase was found in the cervical spinal cord (Figure 7C). On the contrary, in SOD1-G93A mice, *linc-Enc1* was found to decrease in the hippocampus, prefrontal cortex, and increase in the striatum, cervical, and thoracic spinal cord (Figure 7C).

Lastly, the lncRNAs *Fendrr* presented a significant down-regulation in the prefrontal cortex and a significant upregulation in the cervical spinal cord of 8 weeks old SOD1-G93A mice, whilst in the other areas only a trend of change was present (Figure 8A). It resulted altered in different areas of mice aged 18 weeks, specifically it resulted down-regulated in the hippocampus and up-regulated in the thoracic spinal cord (Figure 8B). Concerning *Fendrr*, an upregulation was found in murine aging in the cervical spinal cord in both WT and SOD1G93A mice, whereas a decrease was found in the thoracic spinal cord and an increase in the lumbar one only in WT mice (Figure 8C).

### 3.5. The Expression of Human Homologues of Investigated lncRNAs in an In Vitro Model of ALS Resulted Significantly Deregulated

**Figure 9:**
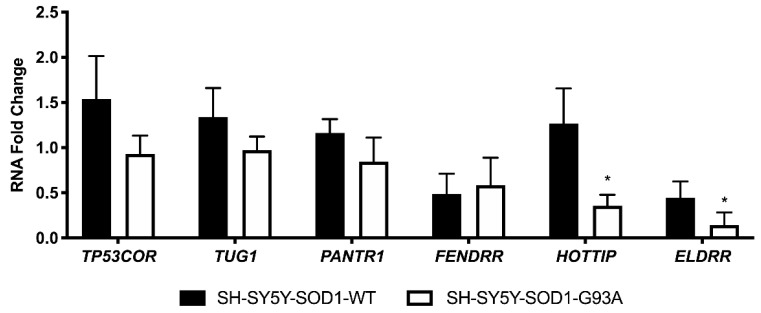
Analysis of human homologues of investigated lncRNAs. RNAs expression levels were evaluated by Real Time-PCR in triplicates in 3 independent experiments (*n* = 9). Data are expressed as mean ± SEM. *p < 0.05 vs. SH-SY5Y-SOD1-WT.

We thus decided to see if our findings could translate to human ALS. Specifically, the human homologues were *TP53COR* (*linc-p21*), *TUG1* (*Tug1*), *PANTR1* (*linc-Brn1a*), *FENDRR* (*Fendrr*), *HOTTIP* (*Hottip*), and *ELDRR* (*Eldrr*) [15]. Their expression was analyzed in a human in-vitro model of the disease, specifically in the human neuroblastoma cell line SH-SY5Y stably transfected with the SOD1 gene either WT or carrying the G93A mutation [29]. Remarkably, changes in the lncRNAs expression were observed, with a tendency to be down-regulated by the mutation (Figure 9). Specifically, *HOTTIP* and *ELDRR* appear to be the most significantly down-regulated (Figure 9).

## 4. Discussion

ALS is a devastating neurodegenerative disease characterized by the progressive loss of upper and lower motor neurons, comprised of both sporadic (90% of cases) and familial (10% of cases) forms of the disease [2]. In the complex picture of disease modifying mechanisms, RNA metabolism and epigenetic functions are proving to be central in the development of the pathogenesis, with a strong implication for non-coding molecules such as lncRNAs [5,6,8]. The possibility to develop therapeutic strategies is one of the main reasons for RNA investigation in ALS, as gene therapy is showing great promise in the correction of numerous ALS dysfunctions [2].

For these reasons, we decided to investigate the deregulated expression of a panel of lncRNAs (*linc-Enc1*, *linc–Brn1a*, *linc–Brn1b*, *linc-p21*, *Hottip*, *Tug1*, *Eldrr*, and *Fendrr*) implicated in neural stem cells differentiation and oncogenesis [14,15,16,17,18,19,20,21,22,23,24,25,26,30], in a murine familial model of ALS (the SOD1-G93A mouse) at a pre-symptomatic and symptomatic stage of the disease. We obtained distinct and specific results for each lncRNAs, some being more deregulated in the ALS model than others, with a precise age-related and area-specific deregulation. Indeed, these results could give further insights into the role of the investigated lncRNAs in ALS pathogenesis along with their possible implication in aging. Even if these lncRNAs are known for their essential role in development, their implication in oncogenesis and late-stage disease suggests a role for the molecules also in adult tissues, and it was thus relevant to analyze how they resulted differentially expressed at different stages of murine lifespan. Moreover, aging and ALS are processes tightly related, as normal aging affects the individual cellular components of the lower motor unit, in a pattern similar to that of ALS [34]. Indeed, transcriptomic analysis of the spinal cord of SOD1-G93A or naturally aged mice highlighted an overlap between the two processes, with 90% of aged spinal cord transcripts also found upregulated in ALS [35].

The lncRNA which appears to have the most profound implications in ALS seems to be *linc-p21*. This lncRNA serves as a repressor in p53-dependent transcriptional responses, and indeed its inhibition affects the expression of hundreds of gene targets normally repressed by p53 [32]. Interestingly, *linc-p21* increases in the most involved ALS tissue, the spinal cord, whereas it decreases in all central brain areas of symptomatic mice. Its upregulation in the spinal cord could be responsible of the high cell death and apoptosis present in the tissue. Indeed, *p53* and *p21* are significantly upregulated in symptomatic SOD1-G93A mice, indicating a deregulation in the *p53/linc-p21/p21* pathway. On the contrary, in the brain we speculate for a possible compensatory mechanism. A different mechanism could be exerted by the lncRNA *Tug1*, which was found upregulated in the lumbar spinal cord of SOD1-G93A mice at both 8 and 18 weeks of age. Indeed, in this case there could be an involvement of an inflammatory mechanism, as downregulation of *Tug1* was found to inhibit the expression of *Tril/Tlr4* inflammatory signaling in the spinal cord [33]. In our model, we found an opposite increased expression of *Tril/Tlr4,* indicating an on-going inflammatory phenomenon. It has been reported that degenerative motor neurons and astrocytes release misfolded proteins as SOD1 in ALS, which activate microglia through CD14, Tlr2, and Tlr4. Direct evidence provides a relevant correlation between the intensity of microglial activation and severity of clinical motor neuron deficits [36]. Based on this evidence, the modulation of Tug1 expression could regulate the effect of microglial activation through Tril/Tlr4 pathways decelerating the motor neuron degeneration and extending the lifespan animals.

Interesting evidence of the possible implication of neuronal stem cells’ aberrant development and neurogenesis in ALS came from the investigation of two lncRNAs, *linc-Brn1a* and *linc-Brn1b*, found to be important in stemness maintenance [15]. Interestingly, we found a predominant deregulation in the pre-symptomatic mice, suggesting they could be relevant in early phases of CNS development in SOD1-G93A mice. Both lncRNAs resulted downregulated in prefrontal cortex, and as these two lncRNAs are involved in cortex development, their downregulation in this area is interesting and could be relevant to the cortex thinning observed in ALS [30,37]. Their downregulation in the hippocampus could also be relevant for the presence of the sub-granular zone in this area, as studies in demonstrated a decreased proliferation in this area in patients with ALS [38].

A reduced, although present, deregulation was found for *Hottip, Eldrr, Linc-Enc1*, and *Fendrr*. *Hottip* resulted significantly down-regulated in the cerebellum, mesencephalon, and lumbar spinal cord, whereas it resulted up-regulated in the cervical and thoracic spinal cord. Interestingly, it was found to bind miR-124, which was found upregulated in the brain of SOD1-G93A mice [9,25]. An aberrant sponging mechanism could be responsible of the upregulation of the miRNA in response to the lncRNA down-regulation. *Eldrr* resulted especially upregulated in all three areas of the spinal cord in symptomatic mice, and literature evidence specifically links this lncRNA expression to interneurons [30]. Interestingly, an increased activity in interneurons was found in adult sacral spinal cord of SOD1-G93A mice [39]. *Linc-Enc1* resulted in down-regulation in the lumbar spinal cord and prefrontal cortex, with an opposite regulation in the cervical and thoracic spinal cord, whereas *Fendrr* presented significant down-regulation in the prefrontal cortex and a significant upregulation in the cervical spinal cord of 8-week-old SOD1-G93A mice, whilst it resulted in down-regulation in the hippocampus and up-regulation in the thoracic spinal cord of 18-week-old mice.

Overall, a global perturbation of these lncRNAs is present in SOD1-G93A mice, and interestingly this appears to be more significant in the cervical, thoracic, and lumbar spinal cord at 18W of age. Interestingly, this deregulation is not always concordant or significant to the same degree, as the expression of some lncRNAs seems to be affected differently in the different areas (e.g., *linc-Brn1a* and *linc-Brn1b* only in the thoracic and lumbar spinal cord at 8W, *Tug1* only in the lumbar spinal cord or *Hottip,* which is increased in the cervical and thoracic spinal cord but remarkably downregulated in the lumbar spinal cord). It is worth mentioning that as 18W transgenic mice are symptomatic, an overall perturbation in the CNS could be present and thus an altered expression of the lncRNAs as opposed to that of WT mice could be a bystander effect, and thus a further characterization of the lncRNAs mechanisms of action is necessary. Indeed, there is currently no evidence linking these lncRNAs to ALS, so further studies are necessary in order to investigate the possible mechanism in which they are implicated, as they could be identified as disease-modifying agents, biomarkers, or secondary effects of ALS neurodegeneration.

Our results thus provide a clear characterization of the expression of this panel of lncRNAs in transgenic mice as opposed to control, at 8 and 18W of age. These comparative analyses thus highlight which lncRNAs might be more involved in disease pathogenesis and in what area. Moreover, our findings also reflects on the changes in expression that occur between both WT and SOD1-G93A mice at different stages of lifespan. Often the trend of expression change during aging is concordant in WT and diseases mice, but worth mentioning is that the entity of such change (the lncRNAs increase or decrease) is different in WT and SOD1-G93A mice. Moreover, this is not always the case and attention should be given to those lncRNAs and areas where the trend in expression is opposite during aging (e.g., *Eldrr* and *Fendrr* in the thoracic spinal cord).

We then decided to investigate the expression of human homologues in an in-vitro model of ALS, represented by the SH-SY5Y neuroblastoma cell line stably transfected with either the SOD1-WT or the SOD1-G93A mutation. The lncRNAs all presented a trend of downregulation, and especially significant are the down-regulations present in *HOTTIP* and *ELDRR*. The expression deregulation is opposite to that observed in tumors for *PANTR1* and *HOTTIP*, as *PANTR1* is upregulated in glioma and nasopharyngeal carcinoma and *HOTTIP* was found upregulated in oral tongue squamous cell carcinoma and gastric cancer [24,25]. It can be hypothesized that these lncRNAs, even if in different tissues, could play a role in the molecular mechanisms inducing oncogenesis as opposed to neurodegeneration, although further studies are needed to characterize their specific function in the disease. Based also on our observations, we can speculate for a “paradigm” in which the lncRNAs deregulation is opposite in cancer and neurodegeneration. Moreover, although there are differences in the lncRNAs deregulation in murine tissues as opposed to stably transfected SH-SY5Y, this could be due to the differences present in the models itself, especially because they refer to a comparison between in-vivo and in-vitro systems. Moreover, our work is to be referred to as a pilot study, as further follow-up would certainly be necessary to assess the lncRNAs mechanism of action in ALS pathogenesis. Indeed, it would be interesting to investigate the lncRNAs expression also in tissues endogenously expressing SOD1 mutations (including CRISPR knock-in models, iPSCs or post-mortem tissues from ALS patients with the SOD1 mutation).

## 5. Conclusions

In conclusion, our study is the first characterization of a panel of 8 lncRNAs (*linc-Enc1*, *linc–Brn1a*, *linc–Brn1b*, *linc-p21*, *Hottip*, *Tug1*, *Eldrr*, and *Fendrr*) in ALS, indicating a deregulation in all of their expression in specific ALS affected areas or at different stages of the pathogenesis. Although a future functional characterization is needed in order to specifically elucidate the lncRNAs role in the pathogenesis, these molecules could be identified as new disease-modifying agents or candidates in ALS pathogenesis.

## Data Availability

The data presented in this study are available in this article and in Supplementary Table S1.

## Supplementary Materials

The following are available online at https://www.mdpi.com/article/10.3390/biomedicines9070809/s1, Table S1: List of primers used.

## References

[1] Wijesekera L.L., Leigh P.N. (2009). Amyotrophic lateral sclerosis. Orphanet J. Rare Dis..

[2] Kim G., Gautier O., Tassoni-Tsuchida E., Ma X.R., Gitler A.D. (2020). ALS Genetics: Gains, Losses, and Implications for Future Therapies. Neuron.

[3] Deng H.-X., Hentati A., Tainer J., Iqbal Z., Cayabyab A., Hung W.Y., Getzoff E.D., Hu P., Herzfeldt B., Roos R.P. (1993). Amyotrophic lateral sclerosis and structural defects in Cu, Zn superoxide dismutase. Science.

[4] Ishigaki S., Sobue G. (2018). Importance of Functional Loss of FUS in FTLD/ALS. Front. Mol. Biosci..

[5] Gagliardi S., Pandini C., Garofalo M., Bordoni M., Pansarasa O., Cereda C. (2018). Long non coding RNAs and ALS: Still much to do. Non-Coding RNA Res..

[6] Liu E.Y., Cali C.P., Lee E.B. (2017). RNA metabolism in neurodegenerative disease. Dis. Model. Mech..

[7] Butti Z., Patten S.A. (2019). RNA Dysregulation in Amyotrophic Lateral Sclerosis. Front. Genet..

[8] Gagliardi S., Zucca S., Pandini C., Diamanti L., Bordoni M., Sproviero D., Arigoni M., Olivero M., Pansarasa O., Ceroni M. (2018). Long non-coding and coding RNAs characterization in Peripheral Blood Mononuclear Cells and Spinal Cord from Amyotrophic Lateral Sclerosis patients. Sci. Rep..

[9] Marcuzzo S., Bonanno S., Kapetis D., Barzago C., Cavalcante P., D’Alessandro S., Mantegazza R., Bernasconi P. (2015). Up-regulation of neural and cell cycle-related microRNAs in brain of amyotrophic lateral sclerosis mice at late disease stage. Mol. Brain.

[10] Joilin G., Leigh P.N., Newbury S.F., Hafezparast M. (2019). An Overview of MicroRNAs as Biomarkers of ALS. Front. Neurol..

[11] Marcuzzo S., Kapetis D., Mantegazza R., Baggi F., Bonanno S., Barzago C., Cavalcante P., de Rosbo N.K., Bernasconi P. (2014). Altered miRNA expression is associated with neuronal fate in G93A-SOD1 ependymal stem progenitor cells. Exp. Neurol..

[12] Alrafiah A.R. (2018). From Mouse Models to Human Disease: An Approach for Amyotrophic Lateral Sclerosis. In Vivo.

[13] Galán L., Pinedo U., Vela-Souto A., Guerrero-Sola A., Barcia J.A., Gutierrez A.R., Martinez-Martinez A., Jiménez M.S.B., García-Verdugo J.M., Matías-Guiu J. (2011). Subventricular zone in motor neuron disease with frontotemporal dementia. Neurosci. Lett..

[14] Sauvageau M., Goff L., Lodato S., Bonev B., Groff A.F., Gerhardinger C., Sanchez-Gomez D.B., Hacisuleyman E., Li E., Spence M. (2013). Multiple knockout mouse models reveal lincRNAs are required for life and brain development. Elife.

[15] Carelli S., Giallongo T., Rey F., Latorre E., Bordoni M., Mazzucchelli S., Gorio M.C., Pansarasa O., Provenzani A., Cereda C. (2019). HuR interacts with lincBRN1a and lincBRN1b during neuronal stem cells differentiation. RNA Biol..

[16] Houck A.L., Seddighi S., Driver J.A. (2019). At the Crossroads Between Neurodegeneration and Cancer: A Review of Overlapping Biology and Its Implications. Curr. Aging Sci..

[17] Seo J., Park M. (2020). Molecular crosstalk between cancer and neurodegenerative diseases. Cell. Mol. Life Sci..

[18] Lang H.-L., Hu G.-W., Chen Y., Liu Y., Tu W., Lu Y.-M., Wu L., Xu G.-H. (2017). Glioma cells promote angiogenesis through the release of exosomes containing long non-coding RNA POU3F3. Eur. Rev. Med. Pharmacol. Sci..

[19] Li W., Wu X., She W. (2019). LncRNA POU3F3 promotes cancer cell migration and invasion in nasopharyngeal carcinoma by up-regulating TGF-β1. Biosci. Rep..

[20] Luo J., Wang K., Yeh S., Sun Y., Liang L., Xiao Y., Xu W., Niu Y., Cheng L., Maity S.N. (2019). LncRNA-p21 alters the antiandrogen enzalutamide-induced prostate cancer neuroendocrine differentiation via modulating the EZH2/STAT3 signaling. Nat. Commun..

[21] Ao X., Jiang M., Zhou J., Liang H., Xia H., Chen G. (2018). lincRNA-p21 inhibits the progression of non-small cell lung cancer via targeting miR-17-5p. Oncol. Rep..

[22] Fattahi S., Kosari-Monfared M., Golpour M., Emami Z., Ghasemiyan M., Nouri M., Akhavan-Niaki H. (2020). LncRNAs as potential diagnostic and prognostic biomarkers in gastric cancer: A novel approach to personalized medicine. J. Cell. Physiol..

[23] Zhou Q., Hu T., Xu Y. (2019). Anticancer potential of TUG1 knockdown in cisplatin-resistant osteosarcoma through inhibition of MET/Akt signalling. J. Drug Target..

[24] Liu R., Li Z., Song E., Hu P., Yang Q., Hu Y., Liu H., Jin A. (2020). LncRNA HOTTIP enhances human osteogenic BMSCs differentiation via interaction with WDR5 and activation of Wnt/β-catenin signalling pathway. Biochem. Biophys. Res. Commun..

[25] Xiong L., Tang Y., Tang J., Liu Z., Wang X. (2020). Downregulation of lncRNA HOTTIP Suppresses the Proliferation, Migration, and Invasion of Oral Tongue Squamous Cell Carcinoma by Regulation of HMGA2-Mediated Wnt/β-Catenin Pathway. Cancer Biother. Radiopharm..

[26] Li W., Liu J., Zhao H. (2020). Identification of a nomogram based on long non-coding RNA to improve prognosis prediction of esophageal squamous cell carcinoma. Aging.

[27] Xu X., Zhuang C., Wu Z., Qiu H., Feng H., Wu J. (2018). LincRNA-p21 Inhibits Cell Viability and Promotes Cell Apoptosis in Parkinson’s Disease through Activating α-Synuclein Expression. BioMed Res. Int..

[28] Marcuzzo S., Zucca I., Mastropietro A., de Rosbo N.K., Cavalcante P., Tartari S., Bonanno S., Preite L., Mantegazza R., Bernasconi P. (2011). Hind limb muscle atrophy precedes cerebral neuronal degeneration in G93A-SOD1 mouse model of amyotrophic lateral sclerosis: A longitudinal MRI study. Exp. Neurol..

[29] Cova E., Ghiroldi A., Guareschi S., Mazzini G., Gagliardi S., Davin A., Bianchi M., Ceroni M., Cereda C. (2010). G93A SOD1 alters cell cycle in a cellular model of Amyotrophic Lateral Sclerosis. Cell. Signal..

[30] Goff L.A., Groff A.F., Sauvageau M., Trayes-Gibson Z., Sanchez-Gomez D.B., Morse M., Martin R.D., Elcavage L., Liapis S.C., Gonzalez-Celeiro M. (2015). Spatiotemporal expression and transcriptional perturbations by long noncoding RNAs in the mouse brain. Proc. Natl. Acad. Sci. USA.

[31] Yoon J.-H., Abdelmohsen K., Srikantan S., Yang X., Martindale J.L., De S., Huarte M., Zhan M., Becker K., Gorospe M. (2012). LincRNA-p21 Suppresses Target mRNA Translation. Mol. Cell.

[32] Huarte M., Guttman M., Feldser D., Garber M., Koziol M., Kenzelmann-Broz D., Khalil A.M., Zuk O., Amit I., Rabani M. (2010). A Large Intergenic Noncoding RNA Induced by p53 Mediates Global Gene Repression in the p53 Response. Cell.

[33] Jia H., Ma H., Li Z., Chen F., Fang B., Cao X., Chang Y., Qiang Z. (2019). Downregulation of LncRNA TUG1 Inhibited TLR4 Signaling Pathway-Mediated Inflammatory Damage After Spinal Cord Ischemia Reperfusion in Rats via Suppressing TRIL Expression. J. Neuropathol. Exp. Neurol..

[34] A Pandya V., Patani R. (2020). Decoding the relationship between ageing and amyotrophic lateral sclerosis: A cellular perspective. Brain.

[35] Herskovits A.Z., Hunter T.A., Maxwell N., Pereira K., Whittaker C.A., Valdez G., Guarente L.P. (2018). SIRT1 deacetylase in aging-induced neuromuscular degeneration and amyotrophic lateral sclerosis. Aging Cell.

[36] Liu J., Wang F. (2017). Role of Neuroinflammation in Amyotrophic Lateral Sclerosis: Cellular Mechanisms and Therapeutic Implications. Front. Immunol..

[37] Thorns J., Jansma H., Peschel T., Grosskreutz J., Mohammadi B., Dengler R., Münte T.F. (2013). Extent of cortical involvement in amyotrophic lateral sclerosis – an analysis based on cortical thickness. BMC Neurol..

[38] Galán L., Gómez-Pinedo U., Guerrero A., García-Verdugo J.M., Matías-Guiu J. (2017). Amyotrophic lateral sclerosis modifies progenitor neural proliferation in adult classic neurogenic brain niches. BMC Neurol..

[39] Jiang M., Schuster J.E., Fu R., Siddique T., Heckman C. (2009). Progressive Changes in Synaptic Inputs to Motoneurons in Adult Sacral Spinal Cord of a Mouse Model of Amyotrophic Lateral Sclerosis. J. Neurosci..

