# Supplementary material for: LncRNAs Associated with Neuronal Development and Oncogenesis Are Deregulated in SOD1-G93A Murine Model of Amyotrophic Lateral Sclerosis
Source: Biomedicines. 2021 Jul 13;9(7):809. doi: 10.3390/biomedicines9070809 (PMC8301400; doi:10.3390/biomedicines9070809)
Supplement: Supplementary file 1 [file biomedicines-09-00809-s001.zip › SupplementaryMaterials/Supplementary_Table_S1.pdf]

Supplementary Table S1. Primers list

**Primers Mouse:**
m-*linc-Brn1a* FW: CATCGAGGGAGAGGGACAGAG;
m-*linc-Brn1a* REV: CCAAAGCACCATTTCATCACATCAG;
m-*linc-Brn1b* FW: TGCCAGCTTGGCTTGTACTC;
m-*linc-Brn1b* REV: GCTCCCAAAGGTTCCTGTGTC;
m-*linc-Enc1* FW: CCTCCCTGATCTCTTTGCTTCC;
m-*linc-Enc1* REV: CGACCACTGGTTCTGCACTC;
m-*Fendrr* FW: GGCCACAGCGGTCAGTTAC;
m-*Fendrr* REV: TCTGGTGGAGTCAGATCAAACG;
m-*Hottip* FW: GGCTTTGGGCTGCATCTTTG;
m-*Hottip* REV: GTCCTTCCACGACAGGGATAC;
m-*linc-p21* FW: AGTAGGGTGTTGTTCAGTTGGTAG;
m-*linc-p21* REV: TCACAGGTATGAGGTGCAGAC;
m-*Tug1* FW: TGACTGGCCCAGAAGTTGTAAG;
m-*Tug1* REV: GCAAGCAGGTCTGTGAGACTATTC;
m-*Eldrr* FW: GAGCTACCAGGGAGGTGAAG;
m-*Eldrr* REV: AGCTCAAGGGTTCTATGATGAGAG;
m-*p53* FW: CCTCATCCTCCTCCTTCCCA
m-*p53* REV: GGCAGTCATCCAGTCTTCGG
m-*p21* FW: TGAATGGAGACAGAGACCCCA
m-*p21* REV: CATGAGCGCATCGCAATCAC
m-*Tril* FW: AAGGGCCTCCAGGTGGGCCTGGAATCGGAC;
m-*Tril* REV: CTCGTGCTCCTGCCTGGCCCTCAGGCTGCA;
m-*Tlr4* FW: GACAAGGGCCCTGAGGAGGAGGAGAACAAG;
m-*Tlr4* REV: TTATAGCTCAGGTTCAGAATGGAGGAGAGT;
m-*Gapdh* FW: CCAGGGCTGCCATTTGCAGTGGCAAAGTGG;
m-*Gapdh* REV: CCTGGAAGATGGTGATGGGCTTCCCGTTGA.
**Primers Human:**
h-*ELDRR* FW: TCCACAGGCTTAATGCATCTC;
h-*ELDRR* REV: TGCCAAGGTTAAGCAGACAG;
h-*PANTR1* FW: GGTTTTTCCTGTTTCCTGCTC;
h-*PANTR1* REV: GCAGTGATTCTTTGAGGGAAA;
h-*HOTTIP* FW: TTCTGCAGTGAGACCACAGG;
h-*HOTTIP* REV: AACAGTGTGGACAGGGAAGG;

h-*FENDRR* FW: CTGGACGGGAACGCTGGAGT;
h-*FENDRR* REV: TCTCGGCACCACCTTTCGTG.
h-*TUG1* FW: ACTGTGCAGAAGCCCAGAGT;
h-*TUG1* REV: ATATTGCTCTGGGGCAGT;
h-*TP53COR* FW: GGGTGGCTCACTCTTGGC;
h-*TP53COR* REV: TGGCCTTGCCCGGGCTTGTC;
h-*18S*-FW: GGATCACTAGGTGATATCGAGC;
h-*18S*-REV: ACCAGACAAGAGTTTAAGAGATATGTATC;
